# Deep Learning-Aided Automated Pneumonia Detection and Classification Using CXR Scans

**DOI:** 10.1155/2022/7474304

**Published:** 2022-08-04

**Authors:** Deepak Kumar Jain, Tarishi Singh, Praneet Saurabh, Dhananjay Bisen, Neeraj Sahu, Jayant Mishra, Habibur Rahman

**Affiliations:** ^1^Chongqing University of Posts and Telecommunications, Chongqing, China; ^2^Mody University of Science and Technology, Lachhmangarh, Rajasthan, India; ^3^Madhav Institute of Technology and Sciences, Gwalior, M. P., India; ^4^G.H. Raisoni University, Amaravati, Maharashtra, India; ^5^IES University, Bhopal, M.P., India; ^6^Islamic University, Kushtia, Bangladesh

## Abstract

The COVID-19 pandemic has caused a worldwide catastrophe and widespread devastation that reeled almost all countries. The pandemic has mounted pressure on the existing healthcare system and caused panic and desperation. The gold testing standard for COVID-19 detection, reverse transcription-polymerase chain reaction (RT-PCR), has shown its limitations with 70% accuracy, contributing to the incorrect diagnosis that exaggerated the complexities and increased the fatalities. The new variations further pose unseen challenges in terms of their diagnosis and subsequent treatment. The COVID-19 virus heavily impacts the lungs and fills the air sacs with fluid causing pneumonia. Thus, chest X-ray inspection is a viable option if the inspection detects COVID-19-induced pneumonia, hence confirming the exposure of COVID-19. Artificial intelligence and machine learning techniques are capable of examining chest X-rays in order to detect patterns that can confirm the presence of COVID-19-induced pneumonia. This research used CNN and deep learning techniques to detect COVID-19-induced pneumonia from chest X-rays. Transfer learning with fine-tuning ensures that the proposed work successfully classifies COVID-19-induced pneumonia, regular pneumonia, and normal conditions. Xception, Visual Geometry Group 16, and Visual Geometry Group 19 are used to realize transfer learning. The experimental results were promising in terms of precision, recall, F1 score, specificity, false omission rate, false negative rate, false positive rate, and false discovery rate with a COVID-19-induced pneumonia detection accuracy of 98%. Experimental results also revealed that the proposed work has not only correctly identified COVID-19 exposure but also made a distinction between COVID-19-induced pneumonia and regular pneumonia, as the latter is a very common disease, while COVID-19 is more lethal. These results mitigated the concern and overlap in the diagnosis of COVID-19-induced pneumonia and regular pneumonia. With further integrations, it can be employed as a potential standard model in differentiating the various lung-related infections, including COVID-19.

## 1. Introduction

**Figure 1:**
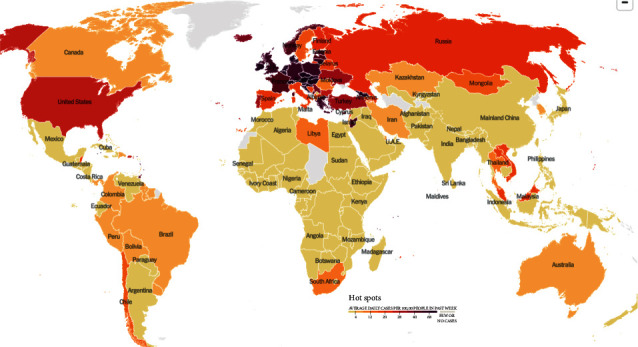
COVID-19 heatmap via NY Times dated 30 November 2021 [3].

**Figure 2:**
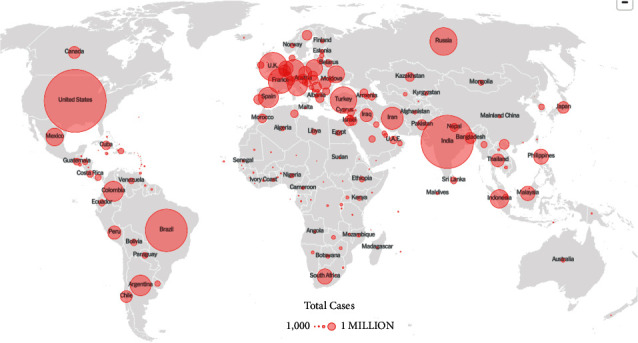
COVID-19 death heatmap via NY Times dated 30 November 2021 [3].

The coronavirus disease (COVID-19) was first identified in the city of Wuhan in China in 2019. As it transcended nations, growing in its impact and severity, the WHO declared it a pandemic. This viral infection peaked at different times in different countries, causing a global calamity. Even in 2022, with each passing day, thousands of new cases are being recorded along with hundreds of deaths. Currently, the total number of COVID-19 cases stands at 26.4 million with 5.25 million casualties worldwide [1]. The affected individual suffers from a series of symptoms and medical complications, depending upon their underlying medical conditions or comorbidities. The current testing methods, such as reverse transcription-polymerase chain reaction (RT-PCR), antigen, and antibody tests, are limited, expensive, and laborious and require a specialized technology, which is often not accessible in remote locations [2]. Moreover, despite being the gold standard, RT-PCR has an accuracy of only 70%. Aside from this, one of the complications arising from COVID-19 exposure includes lung pneumonia, which causes the air sacs in the lung to be filled with fluid. Therefore, investigating chest X-rays becomes essential here, as COVID-19-induced pneumonia can confirm the presence of a COVID-19 infection. However, the symptoms of COVID-19 and other lung inflammatory infections overlap, which makes it prone to misdiagnosis and false positive cases. Figures 1 and 2 illustrate the heatmap of COVID-19 hotspots and the number of deaths due to it [3].

In this regard, artificial intelligence and machine learning techniques have the ability to detect a given pattern from the images. The present paper embraced the ideas of deep leaning, such as Visual Geometry Group 16, 19, and Xception for chest X-rays, to detect COVID-19-induced pneumonia via pretrained models. The proposed model was trained and tested on various architectures to ensure its efficiency and accuracy in classifying chest X-ray images. It used the transfer learning approach with fine-tuning for this purpose. The experimental results demonstrate successful classification of COVID-19-induced pneumonia, normal pneumonia, and normal conditions, thus allaying fear of overlap. These results are encouraging and calculated in terms of precision, recall, F1 score, specificity, false omission rate, false negative rate, false positive rate, and false discovery rate with a COVID-19 detection of almost 98%.

This paper is organized as follows: Section 2 covers the related work; Section 3 represents the proposed work using chest X-ray scans; Section 4 presents the experimental results and analysis with comparison from the current state of art; and Section 5 provides the conclusion.

## 2. Related Work

Artificial intelligence (AI) and big data are of great importance in terms of reacting to the pandemic, along with predicting and analyzing patterns of various strains of viruses.

### 2.1. Background Study

**Figure 3:**
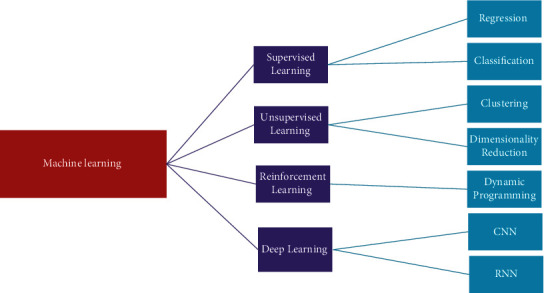
Machine learning types.

The proposed research aims to classify COVID-19-induced pneumonia, regular pneumonia, and healthy patients by using different learning methodologies. Machine learning (ML) is defined as a technique to train a computer to perform tasks that they are not explicitly programmed to do so. It is divided into various types depending on the category of the dataset being utilized as shown in Figure 3. If machine learning is implemented on labeled data, it is known as supervised machine learning, while working on unlabeled data is known as unsupervised machine learning. Similarly, deep learning is also one of the categories based on artificial neural networks. Deep learning architectures like deep neural network, deep belief network, recurrent neural network, and convolutional neural network (CNN) are implemented in the fields of computer vision, natural language processing, and speech recognition. The depth in deep learning signified the artificial neural networks, which are inspired by the human brain, comprised of neurons too which ideally mimic the learning abilities of a human being. The implementation of the suitable machine learning methods and the selection of dataset were the crucial steps when training the model. On the data, various preprocesses, rescaling, and other steps were carried out to make feature extraction easy. In this context, deep learning is preferred, and it reduced the dependency on features by utilizing nonlinear layers for feature extraction [4]. By using sequential layers, it made hierarchical selection based on what features best represented the data, rather than doing a manual feature selection.

The clinical symptoms of COVID-19 comprise fever, dry cough, tiredness, respiratory distress, and much more, which are like that of bronchopneumonia [5, 6]. Medical images like chest X-rays are readily available, and chest X-rays of most COVID-19 cases are bilateral, round-glass opacities with a posterior distribution or peripheral, multifocal, and do remain mainly in the lower lobe of the lung in the early stages [7–9]. Thereafter, it progresses and does pulmonary consolidation in the later stages [10, 11]. Nevertheless, chest X-rays can help in the detection of suspected COVID-19 cases and conditions after it. However, this may lead to ambiguities in the diagnosis of the patient as the chest X-ray images can be identical to multiple other lung diseases, thus requiring further clinical corelation analysis. Such inaccurate diagnoses can lead to common pneumonia being confused with COVID-19 and can cause panic, cost, and unnecessary exposure with COVID-19-positive patients and can be fatal.

### 2.2. Transfer Learning

Data dependency is one of the most complicated issues in deep learning, where adequate training involves a large amount of knowledge to help the network understand data patterns. In the sense of deep learning, all training and testing data are presumed to have the same distribution and feature space. In fact, appropriate training data can exist in one area, while the task of classification is carried out in another. Additionally, if the distribution of data shifts to the target domain, a complete reconstruction of the classification network with the newly collected training dataset is necessary. The CNN models based on transfer learning have advantages such as limited preprocessing of the data, faster learning time, and lower time complexity by reducing the irrelevant parameters. Also, it worked well on the limited dataset, making it ideal for the task of classification of medical images [12].

#### 2.2.1. Visual Geometry Group 19 and Visual Geometry Group 16

Visual geometry group network (VGGNet) is a CNN architecture that focuses on the effect of the convolutional neural network depth on its accuracy [13]. Both versions, namely, VGGNet 19 and VGGNet 16, comprised of two fully connected layers along with 4,096 channels each, which in turn were followed by yet another fully connected layer comprising of 1,000 channels in order to obtain 1,000 labels. The softmax activation function is utilized by the last fully connected layer for classification purposes.

VGG16 is a 16 multilayered architecture proposed by the visual geometry group laboratory at the University of Oxford. It is one of the more widely used architectures because it is composed of 138 million parameters. VGG16 is comprised of 13 convolutional and five maxpool layers. The convolutional layers contained 64 channels as input and output in the dimensions of 224 × 224 × 64.

The ImageNet database is comprised of a fixed input size of 224 × 224 and RGB channels, so the input can be defined as a tensor of (224, 224, 3), which can process the image input up to 1,000 vectors. The vector represented as $\hat{x}$ shows the classification probability for the given class in

(1) $$\begin{array}{c} \hat{x}=\;\left[\begin{array}{c} \hat{x_{0}}\\ \hat{x_{1}}\\ \hat{x_{2}}\\ \hat{x_{3}}\\ .\\ .\\ .\\ \hat{x_{999}} \end{array}\right]. \end{array}$$

The vector in equation (2) presents classification probability for the relevant class. For example, the model predicts a probability of 0.1 for class 0, probability of 0.05 for class 1, probability of 0.05 for class 2, probability of 0.03 for class 3, probability of 0.72 for class 780, and a probability of 0.05 for class 999, while 0 is assigned to the rest of the classes. Thus, the classification vector $\hat{x}$ can be redefined given as follows:

(2) $$\begin{array}{c} \hat{x}=\;\left[\begin{array}{c} \hat{x_{0}}=0.1\\ 0.05\\ 0.05\\ 0.03\\ .\\ .\\ .\\ \hat{x_{780}}=0.72\;\\ .\\ .\\ \hat{x_{999}}=0.05 \end{array}\right]. \end{array}$$

Softmax function is used in equation (1) to ensure that the probabilities add up to 1, which is presented in the following equation:

(3) $$\begin{array}{c} R\left(x=p|\theta^{k}\right)=\;\frac{e^{\theta^{k}}}{\sum_{p=0}^{n}e{\;}^{\theta {\;}_{n}^{k}}}, \end{array}$$

where *θ*= *Y*_0 _*X*_0_+ *Y*_1 _*X*_1_+ … + *Y*_*n* _*X*_*n*_ can be written as shown in the following equation:

(4) $$\begin{array}{c} =\overset{n}{\underset{k=0\;}{\sum }}Y_{k\;}X_{k},\\ =Y^{T}X. \end{array}$$

Now, selecting 5 most probable candidates, the ground truth vector is defined in the following equation:

(5) $$\begin{array}{c} CV=\;\left[\begin{array}{c} 780\\ 0\\ 1\\ 2\\ 999 \end{array}\right],\\ GV=\;\left[\begin{array}{c} GV_{0}\\ GV_{1}\\ GV_{2} \end{array}\right]=\left[\begin{array}{c} 780\\ 2\\ 999 \end{array}\right]. \end{array}$$

Then, the error function “*E*” is calculated and given in the following equation:

(6) $$\begin{array}{c} E=\;\frac{1}{m}\sum_{n}\min_{k}\;d\;\left(CV_{i},GV_{n}\right), \end{array}$$

where *d* = 0; if *CV*_*i*_=*GV*_*n*_, else *d* = 1.

Therefore, the loss function for this particular example is given in the following equation:

(7) $$\begin{array}{c} E=\;\frac{1}{3}\left[\min_{k}d\left(CV_{i},\;GV_{1\;}\right)+\min_{k}d\left(CV_{i},\;GV_{2\;}\right)+\min_{k}d\left(CV_{i},\;GV_{3\;}\right)\right]. \end{array}$$

#### 2.2.2. Xception

**Figure 4:**
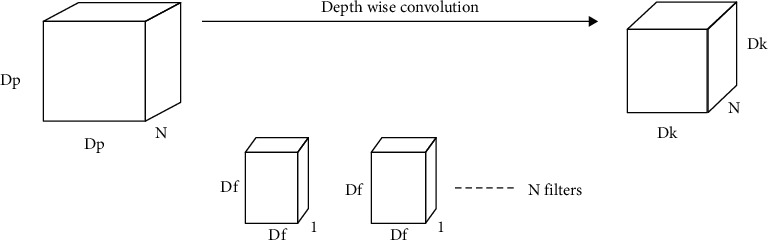
Depth-wise convolution.

**Figure 5:**
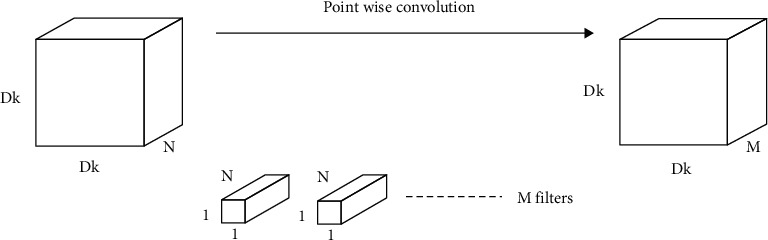
Point-wise convolution.

The Xception architecture is based on the ImageNet database [14], like VGGNet. This architecture is comprised of 36 layered deep separable convolutional layers for feature extraction. Xception functions on a specific type of CNN are known as the depth-wise separable CNN. The properties of such a network included the use of a fewer number of parameters in order to reduce the chances of overfitting, and they became more compatible and computationally cheaper because of their less complex nature. The process of depth-wise separable CNN is divided into depth-wise convolution and pointwise convolution, as shown in Figures 4 and 5.

The depth-wise operation comprised of a convolution being applied to a single channel at a time unlike the CNN operation where it is applied at all “*N*” channels.

Given the *N* channels of input, the filter/kernel size for depth-wise operation is defined as *Df* ∗ *Df* ∗ 1; the output size is summarized as *Dk* ∗ *Dk* ∗ *N*. A single convolutional operation required multiplication of *Df* ∗ *Df* across *N* channels giving the total number of multiplications as *N* ∗ *Dk* ∗ *Dk* ∗ *Dp* ∗ *Dp*; this can be summarized as **N ∗ Df**^**2**^** ∗ Dk**^**2**^. For point-wise operation, a single convolution consists of 1 ∗ *N* multiplication, also written as *Dk* ∗ *Dk* times.

Therefore, the total number of multiplications is “*N* ∗ *Dk* ∗ *Dk* ∗ *M,*” where *M* is the total number of filters. Hence, total number of multiplications in point-wise convolutional operation became **N ∗ Dk**^**2**^** ∗ M**. The overall operation is presented in the following equation:

(8) $$\begin{array}{c} \text{total multiplications }=\text{ depth}-\text{wise operation }+\text{ point}-\text{wise operation}\\ =\left(N\times Df^{2}\times Dk^{2}\right)+\left(N\times Dk^{2}\times M\right),\\ =N\times Dk^{2}\times \left(Df^{2}+M\right). \end{array}$$

### 2.3. Literature Review

AI and deep learning-based detection techniques via medical imaging are gaining popularity because of their promising results in various medical fields. Additionally, there is no lack of data available for training various machine learning-based models. Further, transfer learning has eased this process significantly through the usage of pretrained models that use a lesser number of images in retaining the learned information and detecting it with greater accuracy. Various researchers have used AI- and CNN-based techniques to find the presence of brain tumors [15], lesions [16], breast cancer [17], etc., as summarized in Table 1. CNN is used on CT scans to identify the nature of the malignant pulmonary nodes [18], along with pneumonia via chest imaging scans [19, 20]. Chouhan et al. [21] implemented deep learning architecture to detect the presence of pneumonia using the AlexNet, DenseNet121, InceptionV3, ResNet18, and GoogLeNet neural networks. Mahmud et al. [22] proposed an efficient technique for training an efficient deep neural network using relevant, available X-ray images so that the learned parameters could be utilized to detect COVID-19 instances even though the available COVID-19 X-ray dataset contained fewer images. Based on deep learning algorithms and unique features, Gu et al. [23] proposed an automated bacterial or viral pneumonia diagnostic approach to chest radiographs. On chest CT examinations, Li et al. [24] utilized a deep learning technique to identify COVID-19 and community-acquired pneumonia (CAP) using a deep learning method. Finally, a powerful deep learning model was developed for identifying COVID-19 and CAP from chest CT scans. These findings have shown a convolutional network model-based machine-learning strategy to discriminate COVID-19 from CAP. Rajpurkar et al. [25] demonstrated CheXNeXt, a deep learning algorithm that detected various thoracic diseases in frontal-view chest radiographs, as well as practicing board-certified radiologists. They developed and evaluated a deep learning system that accurately detected clinically significant abnormalities in chest radiographs, on par with expert radiologists. Chowdhury et al. [26] proposed a deep CNN-based transfer learning method for automatically identifying COVID-19 pneumonia. The authors trained, validated, and assessed eight popular and previously reported effective CNN-based deep learning algorithms for distinguishing pneumonia patients from normal ones, using chest X-ray pictures. When image augmentation was not used, CheXNet, a DenseNet descendant, outperformed the other networks. Thereafter, Liang and Zheng [27] demonstrated an automated diagnostic method that differentiated between normal and pneumonia-affected children's chest X-ray pictures. They built a new network architecture with residual components to better comprehend the effective textural properties of the lung tissue. There were 49 convolutional layers in the network, as well as the ReLU activation, one global average pooling layer, and two dense layers. Ho and Gwak [28] offered a unique framework for integrating numerous characteristics from both shallow and deep features. After completing extensive tests, representative and discriminative characteristics were developed to differentiate 14 diseases from the public chest X-ray dataset. The use of deep learning (DL) algorithms to analyze lung ultrasonography (LUS) images was studied by Roy et al. [29]. The authors provided a new and completely annotated dataset of LUS images obtained from several Italian hospitals, with labels representing the illness severity on a frame-by-frame, video-by-video, and pixel-by-pixel basis. Table 1 presents the summary of some of the AI and machine learning techniques used for the detection of different diseases.

After reviewing the current state of the art, it can be confirmed that AI and machine learning techniques are used to investigate chest X-rays to ascertain the presence of any disease, including COVID-19 infection. Further, there is currently no dearth of datasets to train and test the results. This paves the way to develop a mechanism to detect COVID-19 infection.

## 3. Proposed Methodology

**Figure 6:**
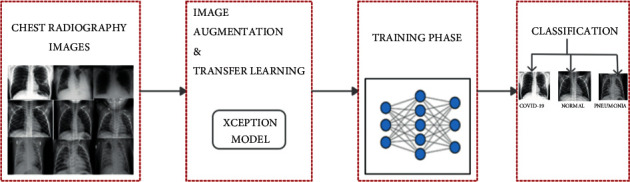
Proposed work architecture.

The conventional methodologies in COVID-19 testing like the antigens, antibodies, and RT-PCR are associated with high-end medical infrastructure and cost and suffered from delays. Also, on the qualitative front, it bears poor detection accuracy and reported only 70% accuracy. As lung inflammation and infection are common across various COVID-19 cases, the use of a chest X-ray is considered a viable option. This section introduces a model based on CNN and deep learning techniques to detect COVID-19-induced pneumonia using chest X-rays. This work also made a distinction between COVID-19-induced pneumonia and regular pneumonia as the latter is a common disease, and one should not confuse it with the former, which is more lethal and fatal. The proposed model utilizes deep learning, transfer learning, and a pretrained model Xception on various training and testing ratios and consists of 4 phases presented in Figure 6.

**Figure 7:**
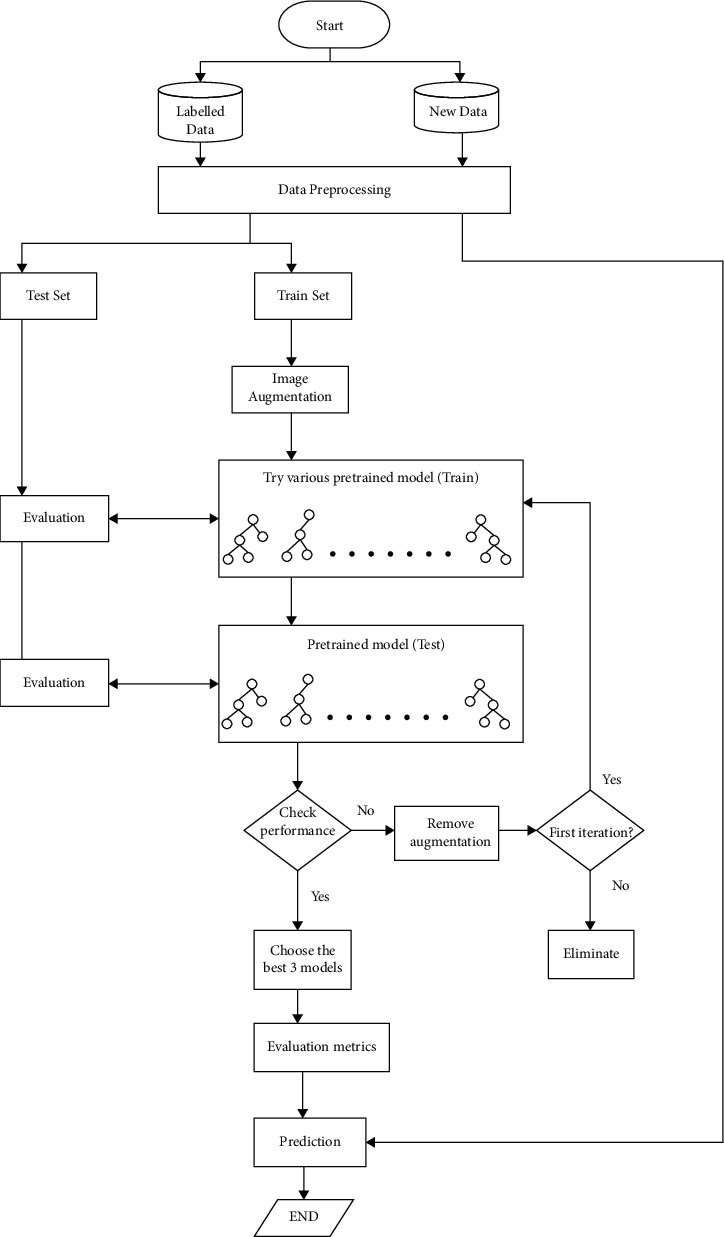
Proposed workflow.

The proposed workflow model of this network is demonstrated in Figure 7, where firstly, the acquired labeled data are preprocessed, then split into 80 : 20, 70 : 30, and 60 : 40 ratios of test and train, applied with image augmentation properties. Furthermore, the images are subjected to various pretrained models such as VGG16, VGG19, and Xception that involve transfer learning techniques. If the accuracy is not adequate in one ratio, the images are trained again with a different set of ratios. The models are chosen based on their accuracy and other evaluations. The chosen models are included in this study with their classification performance.

### 3.1. Phase I: Dataset Description

Medical scans in form of chest X-rays are essential for a computerized diagnosis. This work uses a curated dataset for COVID-19 posterior-anterior chest radiography images (X-ray) proposed by Sait et al. [30].

This dataset compiled 15 publicly available datasets and removed unwanted properties like noise, pixelation, and compression. It also removed unwanted images such as scans with medical implants, washed-out images, images with a side view, CT (sliced) images, images with aspect ratio distortion like cropping and zooming, rotated images, and images with annotations. From this dataset, 1,281 images of COVID-19, 1,300 images of pneumonia, and 1,481 normal images were selected randomly for training and validation purposes.

**Figure 8:**
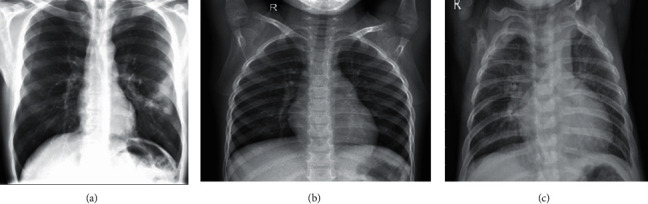
Chest X-ray data [30]: (a) COVID-19 positive, (b) normal healthy lung, and (c) regular pneumonia.

The original images were X-ray scans with dimensions of 450 × 456 pixels. These scans are represented in Figure 8.  Table 2 shows that the images were divided into three classes with various training and validation distributions. These scans were further subjected to augmentation properties depending on the accuracy they provided.

### 3.2. Phase II: Preprocessing

Image preprocessing involves balancing the elements of an image in accordance with the need of the proposed model, which can greatly affect accuracy and prediction. The input scans obtained from the dataset were rescaled and reshaped to the desired size (224 × 224), along with other augmentation properties before training the model. Image augmentation properties included removal of unwanted noise, pixelation, medical implants, compression, zooming, cropping, and images with labels. The scans were also rotated, shifted, and improved in sheerness and brightness. Image augmentation highly influenced a model's training time and performance. The detailed operations that were carried out are provided in Table 3.

### 3.3. Phase III: Training and Validation

The augmented images were trained in CNN, Xception, VGG19, and VGG16 with the same properties shown in Table 4. The Xception model provided the highest accuracy and the shortest time compared with the other two models. The images were split in a ratio of 80 : 20, 70 : 30, and 60 : 40 for training and validation. They were further split into three classes: “COVID-19,” “normal,” and “regular pneumonia.”

### 3.4. Phase IV: Results and Classification

**Figure 9:**
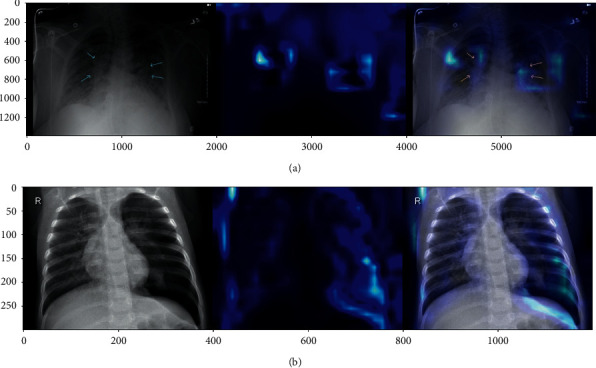
(a) Heatmap COVID-19-induced pneumonia and (b) heatmap regular pneumonia.

The custom-built CNN model and the pretrained models (Xception, VGG19, and VGG16) were tested for 15 epochs with 800 steps each. The experiments were carried out on three train and test ratio combinations of 80 : 20, 70 : 30, and 60 : 40, wherein 80% of the total images were for training purposes and 20 for testing. This paper discusses the results obtained on the 80 : 20 ratio, as it is the most efficient one when compared with the other two ratios and was performed on the Google collaboratory platform with the GPU runtime as provided. Further, along with the performance, the detections are displayed using a confusion matrix, and the classification reports along with the detection of COVID-19-induced and regular pneumonia are shown through the heatmaps in Figures 9(a) and 9(b).

## 4. Experimentations and Result Analysis

This section presents the experimental results of the proposed model with CNN for their comparison against the pretrained models. The CNN method is built with each of the custom layers being defined well. The difference between CNN and the pretrained models is that the latter utilize transfer learning, while CNN does not. The image augmentation and training parameters are the same in both kinds of models.

### 4.1. Model-Wise Experimentation

**Figure 10:**
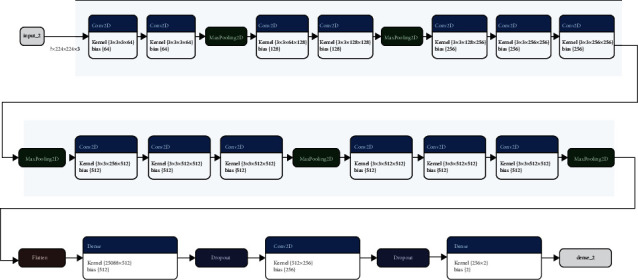
CNN layer-wise architecture.

The layers of the CNN model are shown in Figure 10; the model consists of several convolutional layers followed by max-pooling, flatten, dropout, and dense layers. The classification results of CNN were calculated through a confusion matrix, and other matrices like the F1 score, recall, precision, sensitivity, specificity, FOR, FNR, FPR, and FDR.

**Figure 11:**
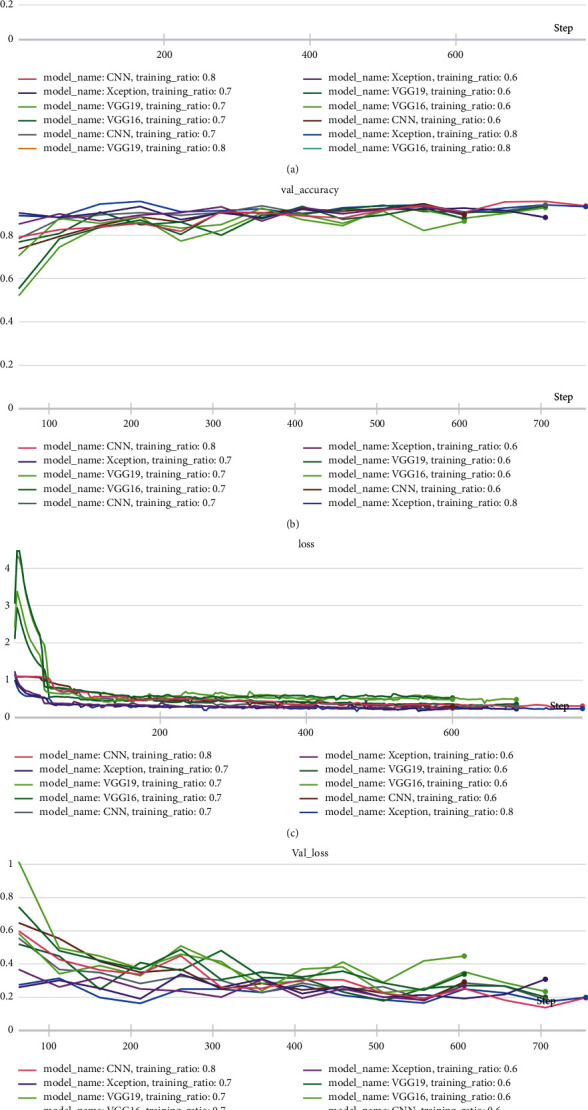
(a) Training accuracy of the proposed networks on various training and testing ratios, (b) validation accuracy of the proposed networks on various training and testing ratios, (c) training loss of the proposed networks on various training and testing ratios, and (d) validation loss of the proposed networks on various training and testing ratios.

These experiments were conducted for formulating a model that can accurately distinguish between COVID-19-induced pneumonia, regular pneumonia, and healthy lungs.  Table 5 and Figures 11(a)–11(d) present the experimental results, using different networks for training and validation accuracy against different training and test set ratios.

**Figure 12:**
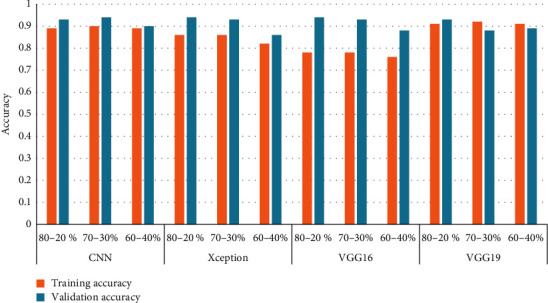
Model-wise comparison of accuracies.

**Figure 13:**
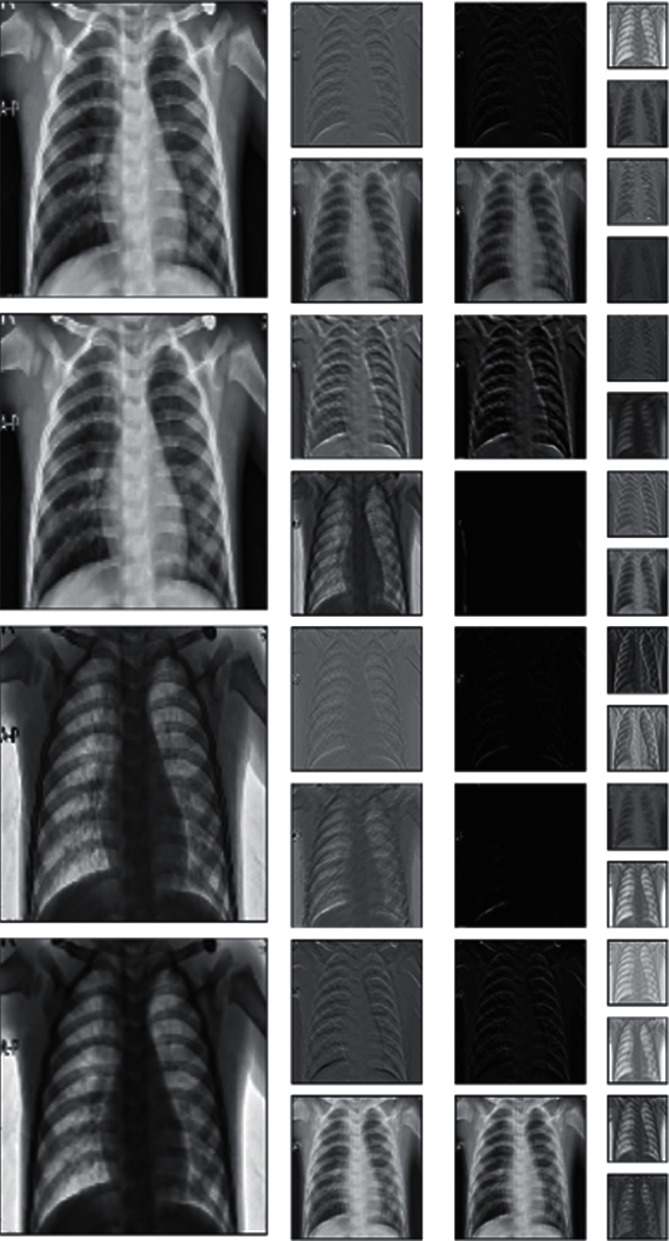
Xception layer-wise processing through 0, 3, 6, and 9 layers.

These experimental results show that the CNN model achieves its best performance using the 80%–20% train-to-test ratio with 2,842 training samples and 1,281 test samples. With this ratio, the CNN reported a training accuracy of 89%, while its validation accuracy remained at 93%, a bit lower than Xception with minimal loss. For the same network, the 70%–30% and 60%–40% train-to-test ratios yielded a training accuracy of 90% and 89%, respectively, and a validation accuracy of 94% and 90%, respectively. These results also provide a comparison to the training and validation accuracy of other networks in the 80%–20%, 70%–30%, and 60%–40% train-to-test combinations. The observations clearly indicate that all networks report a higher training and validation accuracy as the training set size increases.  Figure 12 shows the training and validation accuracy for all models with different train-to-test ratios. This comparison focuses on the 80%–20% train-to-test ratio, as it yields the highest performance for all models when compared to the other train-to-test ratios. The Xception architecture outperformed the other three models with a validation accuracy of 94%, while VGG19 reported the lowest validation accuracy. These experimental results highlight the efficiency of the Xception model over other models, and with aditional integrations, its accuracy and performance can be further improved. Tests of different transfer learning models such as VGG16, VGG19, and Xception revealed that VGG19 reported the highest training accuracy, while Xception and VGG16 presented the highest validation accuracy. Xception also yielded better training accuracy than VGG16. Lastly, the functioning of the proposed method through the distinct layers is shown in Figure 13.

### 4.2. Confusion Matrix Evaluation

A confusion matrix represents various qualitative parameters [31, 32]. Ideally, the true positive rate and the true negative rate should be close to 100% in order to provide correct classification.

Similarly, the false positive and false negative rates should be as close to 0% as possible to reduce the chances of incorrect detection. Different classification matrices are given as follows:

(i) Accuracy: accuracy expresses the number of data instances identified correctly over the total number of data instances given in the following equation:
  (9) $$\begin{array}{c} \frac{\text{TP}+\text{TN}}{\text{TP}+\text{TN}+\text{FP}+\text{FN}}\text{.} \end{array}$$
(ii) Precision/positive predicted value: precision, as shown in equation (10), is the ratio of the positive cases identified correctly to all the positive cases expected.
  (10) $$\begin{array}{c} \frac{\text{TP }}{\left(\text{TP }+\text{ FP}\right)}\text{.} \end{array}$$
(iii) Recall/sensitivity/true positive rate: the instances that are correctly defined as positive cases compared to all the real positive cases are recall, as shown in the following equation:
  (11) $$\begin{array}{c} \frac{\text{TP }}{\left(\text{TP }+\text{ FN}\right)}\text{.} \end{array}$$
(iv) F1 score: the harmonic mean of accuracy and recall is the F1 metric; it is a better metric than accuracy, as illustrated in the following equation:
  (12) $$\begin{array}{c} \frac{2*\left(\text{Precision}*\text{Recall}\right)}{\left(\text{Precision}+\text{Recall}\right)}\text{.} \end{array}$$
(v) Specificity/true negative rate: it is the number of true labels that lie in the class, shown in the following equation:
  (13) $$\begin{array}{c} \frac{\text{ TN}}{\left(\text{TN }+\text{ FP}\right)}\text{.} \end{array}$$
(vi) False discovery rate: the ratio of false positive results to the total of false positive and true positive results observed shown in equation (13).
  (14) $$\begin{array}{c} \frac{\text{ FP}}{\left(\text{FP }+\text{ TP}\right)}\text{.} \end{array}$$
(vii) False negative rate: the error that signified that a particular condition did not hold while it existed, referred in the following equation:
  (15) $$\begin{array}{c} \frac{\text{FN}}{\left(\text{FN }+\text{ TP}\right)}\text{.} \end{array}$$
(viii) False omission rate: the ratio in a test for a condition being probably true provided that the results are deemed as false, illustrated in the following equation:
  (16) $$\begin{array}{c} \frac{\text{FN }}{\left(\text{FN }+\text{ TN}\right)}. \end{array}$$
(ix) False positive rate: the probability that a false value is given wherein a result is declared positive, while its true value is negative, presented in the following equation:
  (17) $$\begin{array}{c} \frac{\text{FP}}{\left(\text{FP }+\text{ TN}\right)}\text{.} \end{array}$$

**Figure 14:**
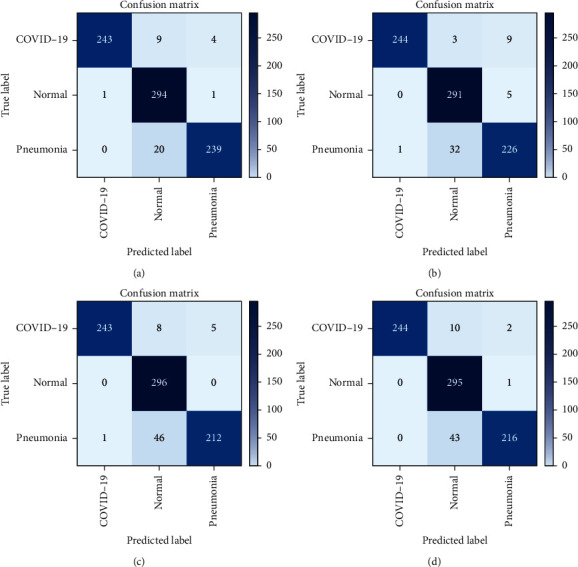
(a) CNN confusion matrix, (b) Xception confusion matrix, (c) VGG16 confusion matrix, and (d) VGG19 confusion matrix.

Confusion matrices of the experimental results are presented in Figures 14(a)–14(d). These results illustrated the experimental findings for CNN, Xception, VGG16, and VGG19. In terms of COVID-19-induced pneumonia diagnosis, true positives are denoted as TP, the number of patients that are accurately diagnosed as COVID-19 positive. True negatives are denoted as TN, the number of patients that are accurately diagnosed as COVID-19 negative. False positives are denoted as FP, the number of patients that are misdiagnosed as COVID-19 positive. False negatives are denoted as FN, the number of patients who are misdiagnosed as COVID-19 negative.  Table 6 demonstrates the confusion matrix evaluation of the three classes by showcasing the training-testing ratio and classifying the images as TP, TN, FP, and FN. Experimental results were calculated for train-to-test ratios like 80 : 20, 70 : 30, and 60 : 40, respectively. The total numbers of validation data were 812, 1,218, and 1,624. Number of images classified in terms of FN, FP, TN, and TP by each model. Out of these data, a majority were reported in the true positive and true negative classes, making them rightly classified. Xception and VGG19 network classified the highest greatest number of images correctly in 80 : 20 ratio and reported the lowest number of images detected wrongly, while successfully detecting 799 images with precision.

Table 7 presents the various performance matrices as shown in equations (9)–(17). These results illustrate the classification report of the three classes—COVID-19, normal, and regular pneumonia—with the total number of images that were correctly detected, incorrectly detected, and not detected at all. Experimental results revealed that COVID-19 detection accuracy in 80 : 20 train-to-test ratio remained as high as 98% for all the network models. This is quite significant when compared to conventional tests used to detect COVID-19 presence. However, for the “normal” category, different networks reported different detection accuracy—96% for CNN, 94% for VGG16 and VGG19, and 97% for Xception—for 80 : 20 train-to-test ratio. Similarly, different networks again yielded different detection rates for “regular pneumonia,” for which detection accuracy for CNN, VGG16, VGG19, and Xception remained at 97%, 94%, 95%, and 96%, respectively, for 80 : 20 train-to-test ratio. Other result matrices followed the same trend, reporting better values for greater training set size.

Experimental results presented in Table 8 illustrate the classification report for each network in three different testing-and-training ratio. Out of all the ratios, the 80 : 20 train-and-test ratio reported the most encouraging results. This training-and-testing set is comprised of a total of 812 images—the COVID-19 class with 256 images, the pneumonia class with 260, and the normal class with 296. In the COVID-19 category, the proposed network CNN reported 243 correctly detected images while unable to detect or wrongly detected 14 images. VGG19 and Xception reported the same number of 244 correctly identified images, and lastly, VGG16 correctly detected 243 images and either wrongly or fail to detect 14 images. Similarly, for the regular pneumonia class, out of the 260 total images, CNN detected 239 images accurately, while 5 were wrongly detected and 20 were undetected.

VGG19 identified 216 scans precisely; however, three were incorrectly detected, and 43 remained undetected. Similarly, VGG16 successfully identified 212 instances, but 5 were incorrectly identified, while 4 were not detected. Xception reported 226 precise detections with 13 wrongly and 33 not detected. Thereafter, for the “normal” category, out of 296 total instances, CNN correctly identified 294 instances, while 29 were wrongly identified and 29 remained undetected. The Xception network correctly identified scans in 291 instances, but 35 were incorrectly identified, and five were not identified. Afterward, VGG19 correctly detected scans in 295 instances, while 53 were incorrectly identified, and one was not detected. Lastly, VGG16 successfully identified scans in 296 instances, while 54 were wrongly detected. There were no instances when a scan remained undetected.

It is evident from these results that Xception performed slightly better compared with the other two pretrained models. With these results, it can be concluded that the Xception network performed efficiently in correctly categorizing X-rays across different training and test ratios, and it is also proficient at detecting various other lung infections. Overall, the experimental results showed that chest X-rays could be used with machine learning techniques to identify the presence of COVID-19 or COVID-19-induced pneumonia. It can also successfully differentiate COVID-19 from regular pneumonia and normal conditions.

## 5. Conclusion

The paper presented a mechanism that embraced the ideas of deep learning, deep neural networks, convolutional neural networks, and transfer learning theories and that successfully identified COVID-19 and COVID-19-induced pneumonia using chest X-rays. The proposed work used transfer learning to report encouraging and accurate experimental results, as it did not require a large dataset. Experimental results also alleviated concern regarding overlap between diagnoses of COVID-19 and regular pneumonia.

The custom CNN and transfer learning architectures trained on various training and testing ratios provide enough evidence in terms of efficiency. The detection accuracy stands at 98% for all the networks. For other combinations, Xception and VGG16 showcase similar results, but Xception can be categorized as better because its loss value is better than other networks. The proposed methodology works effectively and differentiates the two infections (COVID-19/COVID-19-induced pneumonia and regular pneumonia), which can benefit the medical infrastructure with further integrations. The application of artificial intelligence exhibits a lot of scope in the detection and diagnosis of COVID-19 and regular pneumonia by training this model on other lung infections. Therefore, strengthening the dataset in size will increase its efficiency and will amplify its application. Furthermore, other deep learning models like GoogLeNet and AlexNet can apply to chest X-ray datasets to obtain promising results.

## Figures and Tables

**Table 1:** Current state of the art.

Author	Year	Data sets/samples	Sample collection location/source	Network type/technique used	Objective of study	Type of study/outcome of study
Mahmud et al. [22]	2020	Total of 5856 pictures	Guangzhou Medical Center, China and Sylhet Medical College, Bangladesh	CNN	To utilize COVID-19 chest X-rays for efficiently extracting diversified features from varying dilation rates	Detection accuracy:
		1583 normal X-rays, 1493 non-COVID, 2780 bacterial pneumonia				97.4% for COVID-19/normal pneumonia
						96.9% for COVID-19/viral pneumonia
						94.7% for COVID-19/bacterial pneumonia

Gu et al. [23]	2018	JSRT, 241 images and Montgomery County, (MC, 138 images)	Guangzhou Women and Children's Medical Center, China	FCN and DCNN	Deep learning in chest radiography for diagnosis of bacterial and viral childhood pneumonia	Experiments revealed that DCNN with transfer learning extracted features with greater accuracy (0.8048 ± 0.0202) and sensitivity (0.7755 ± 0.0296)
Li et al. [24]	2020	At six medical centers, 4,536 volumetric chest CT examinations (3D) were obtained from 3,506 individuals	6 different hospitals in China	COVID-19 detection neural network (COVNet)	Distinguishing of COVID-19 from community-acquired pneumonia on chest CT using AI	For community-acquired pneumonia, the area under the receiver operating characteristic curve was 0.96 and 0.95, respectively
			(From August 2016 and February 2020)			

Rajpurkar et al. [25]	2018	420 images from 14 different pathologies	Bethesda, Maryland, United States	CheXNeXt algorithm	To diagnosis chest radiograph using deep learning method	The 420 radiographs were labeled by radiologists in an average of 240 minutes, and the algorithm labeled them in 1.5 minutes

Chowdhury et al. [26]	2020	Total 423 images database (1485 pictures of viral pneumonia and 1579 pictures of normal chest X-rays)	Italian Society of Medical and Interventional Radiology, Italy	CNN	Screening of COVID-19 and pneumonia detection using AI	The networks were trained to distinguish between two types of pneumonia. For both methods, the classification accuracy, precision, sensitivity, and specificity were 99.7%, 99.7%, 99.7%, and 99.55% and 97.9%, 97.95%, 97.9%, and 98.8%, respectively

Liang and Zheng [27]	2020	Total 5856 chest X-ray images (training: 5232, testing: 624)	Guangzhou Women and Children's Medical Center, China	CNN	Pediatric pneumonia diagnosis using transfer learning technique with a deep residual network	On a children's pneumonia classification test, the method recall rate is 96.7%, and the F1 score is 92.7%

Ho and Gwak [28]	2019	Total of 112,120 X-ray images (70% training,	ILSVRC2014 dataset	CNN/DenseNet-121 model	CNN-based classification of thoracic disease in chest radiography	In compared to current reference baselines, techniques efficiently used interdependencies among target annotations to produce state-of-the-art classification results of 14 diseases
		10% validation, and 20% testing)				

Roy et al. [29]	2020	There are 58,924 frames in 277 lung ultrasound recordings from 35 individuals	Italian COVID-19 lung ultrasound	CNN	Diagnosis of lung diseases in COVID-19 pandemic using deep learning	A novel deep network based on spatial transformer networks that predicts the illness severity with weakly supervised artefact localization
			Database (ICLUS-DB), Italy			

**Table 2:** Distribution of the dataset.

Classes	80%–20%		70%–30%		60%–40%	
	Training	Validation	Training	Validation	Training	Validation
COVID-19	1,024	256	896	384	768	512
Normal	1,184	296	1,036	444	888	592
Pneumonia	1,040	260	910	390	780	520
Total	3,245	812	2,842	1,218	2,436	1,624

**Table 3:** Image preprocessing details.

S. no.	Operations	Description
1.	Rotation	10° clockwise and anticlockwise
2.	Width shifting	0.1 fraction of the total width
3.	Height shifting	0.1 fraction of the total height
4.	Zooming	0.2% smaller or larger of original image
5.	Rescaling	1/255 multiplied with image channel values to the normalize input
6.	Sheerness	0.2 degrees clockwise and anticlockwise
7.	Brightness	0.25–1.0 shift value

**Table 4:** Training properties.

S. no.	Training parameters	CNN	Xception	VGG19	VGG16
1.	Learning rate	0.001	0.001	0.001	0.001
2.	Batch size	64	64	64	64
3.	Brightness range	[0.25, 1]	[0.25, 1]	[0.25, 1]	[0.25, 1]
4.	Height and width shift range	0.1	0.1	0.1	0.1
5.	Rotation range	10	10	10	10
6.	Share range	0.2	0.2	0.2	0.2
7.	Zoom range	[0.8, 1.2]	[0.8, 1.2]	[0.8, 1.2]	[0.8, 1.2]
5.	Input shape	[224, 224, 3]	[224, 224, 3]	[224, 224, 3]	[224, 224, 3]
6.	Loss function	Categorical cross-entropy	Categorical cross-entropy	Categorical cross-entropy	Categorical cross-entropy
7.	Rescale	1/225	1/225	1/225	1/225
8.	Epoch	15	15	15	15
9.	Training set	80%, 70%, and 60%	80%, 70%, and 60%	80%, 70%, and 60%	80%, 70%, and 60%

**Table 5:** Training and validation accuracy of proposed networks.

Network	Training and test ratio (%)	Training accuracy	Validation accuracy
CNN	80–20	0.89	0.93
	70–30	0.90	0.94
	60–40	0.89	0.90

Xception	80–20	0.86	0.94
	70–30	0.86	0.93
	60–40	0.82	0.86

VGG16	80-20	0.78	0.94
	70–30	0.78	0.93
	60–40	0.76	0.88

VGG19	80-20	0.91	0.93
	70–30	0.92	0.88
	60–40	0.91	0.89

**Table 6:** Total number of FN, FP, TN, and TP for each of the three classes.

Model name	Training and test ratio	COVID-19				Normal				Pneumonia			
		FN	FP	TN	TP	FN	FP	TN	TP	FN	FP	TN	TP
CNN	80–20%	13	2	554	243	1	30	486	295	22	4	548	238
	70–30%	29	2	832	355	37	14	760	407	8	58	770	382
	60–40%	30	13	1099	482	15	58	974	577	45	19	1085	475

VGG16	80–20%	13	1	555	243	0	47	469	296	40	5	547	220
	70–30%	23	6	828	361	4	60	714	440	49	10	818	341
	60–40%	46	10	1102	466	0	145	887	592	113	4	1100	407

VGG19	80-20%	12	1	555	244	0	49	467	296	40	2	550	220
	70–30%	24	5	829	360	9	70	704	435	62	20	808	328
	60–40%	38	5	1107	474	9	86	946	583	74	30	1074	446

Xception	80-20%	12	2	554	244	1	25	491	295	23	9	543	237
	70–30%	27	3	831	357	7	51	723	437	49	29	799	341
	60–40%	26	8	1104	486	0	89	943	592	83	12	1092	437

**Table 7:** Precision, recall, F1 score, and support of all three classes.

Model name		CNN			VGG16			VGG19			Xception		
Training and test ratio		80–20%	70–30%	60–40%	80–20%	70–30%	60–40%	80–20%	70–30%	60–40%	80–20%	70–30%	60–40%
COVID-19	Accuracy	0.98	0.97	0.97	0.98	0.98	0.97	0.98	0.98	0.97	0.98	0.98	0.98
	F1 score	0.97	0.96	0.96	0.97	0.96	0.94	0.97	0.96	0.96	0.97	0.96	0.97
	FDR	0.01	0.01	0.03	0.00	0.02	0.02	0.00	0.01	0.01	0.01	0.01	0.02
	FNR	0.87	0.94	0.70	0.93	0.79	0.82	0.92	0.83	0.88	0.86	0.90	0.76
	FOR	0.02	0.03	0.03	0.02	0.03	0.04	0.02	0.03	0.03	0.02	0.03	0.02
	FPR	0.00	0.00	0.01	0.00	0.01	0.01	0.00	0.01	0.00	0.00	0.00	0.01
	Precision	0.99	0.99	0.97	1.00	0.98	0.98	1.00	0.99	0.99	0.99	0.99	0.98
	Recall	0.95	0.92	0.94	0.95	0.94	0.91	0.95	0.94	0.93	0.95	0.93	0.95
	Sensitivity	0.95	0.92	0.94	0.95	0.94	0.91	0.95	0.94	0.93	0.95	0.93	0.95
	Specificity	0.44	0.43	0.43	0.44	0.43	0.42	0.44	0.43	0.43	0.44	0.43	0.44

Normal	Accuracy	0.96	0.96	0.96	0.94	0.95	0.91	0.94	0.94	0.94	0.97	0.95	0.95
	F1 score	0.95	0.94	0.94	0.93	0.93	0.89	0.92	0.92	0.92	0.96	0.94	0.93
	FDR	0.09	0.03	0.09	0.14	0.12	0.20	0.14	0.14	0.13	0.08	0.10	0.13
	FNR	0.03	0.73	0.21	0.00	0.06	0.00	0.00	0.11	0.09	0.04	0.12	0.00
	FOR	0.00	0.05	0.02	0.00	0.01	0.00	0.00	0.01	0.01	0.00	0.01	0.00
	FPR	0.06	0.02	0.06	0.09	0.08	0.14	0.09	0.09	0.08	0.05	0.07	0.09
	Precision	0.91	0.97	0.91	0.86	0.88	0.80	0.86	0.86	0.87	0.92	0.90	0.87
	Recall	1.00	0.92	0.97	1.00	0.99	1.00	1.00	0.98	0.98	1.00	0.98	1.00
	Sensitivity	1.00	0.92	0.97	1.00	0.99	1.00	1.00	0.98	0.98	1.00	0.98	1.00
	Specificity	0.57	0.53	0.56	0.57	0.57	0.57	0.57	0.56	0.56	0.57	0.56	0.57

Pneumonia	Accuracy	0.97	0.95	0.96	0.94	0.95	0.93	0.95	0.93	0.94	0.96	0.94	0.94
	F1 score	0.95	0.92	0.94	0.91	0.92	0.87	0.91	0.89	0.90	0.94	0.90	0.90
	FDR	0.02	0.13	0.04	0.02	0.03	0.01	0.01	0.06	0.06	0.04	0.08	0.03
	FNR	0.85	0.12	0.70	0.89	0.83	0.97	0.95	0.76	0.71	0.72	0.63	0.87
	FOR	0.04	0.01	0.04	0.07	0.06	0.09	0.07	0.07	0.06	0.04	0.06	0.07
	FPR	0.01	0.07	0.02	0.01	0.01	0.00	0.00	0.02	0.03	0.02	0.04	0.01
	Precision	0.98	0.87	0.96	0.98	0.97	0.99	0.99	0.94	0.94	0.96	0.92	0.97
	Recall	0.92	0.98	0.91	0.85	0.87	0.78	0.85	0.84	0.86	0.91	0.87	0.84
	Sensitivity	0.92	0.98	0.91	0.85	0.87	0.78	0.85	0.84	0.86	0.91	0.87	0.84
	Specificity	0.43	0.46	0.43	0.40	0.41	0.37	0.40	0.40	0.40	0.43	0.41	0.40

**Table 8:** Correctly, incorrectly, and not detected among the three classes.

Network	Training and test ratio	COVID-19			Regular pneumonia			Normal		
		Correctly detected	Incorrectly detected	Not detected	Correctly detected	Incorrectly detected	Not detected	Correctly detected	Incorrectly detected	Not detected
CNN	80–20%	243	1	13	239	5	20	294	29	29
	70–30%	335	3	29	378	54	12	409	19	35
	60–40%	482	14	30	480	16	40	576	56	16

Xception	80–20%	244	1	12	226	13	33	291	35	5
	70–30%	357	3	27	343	33	47	433	50	11
	60–40%	486	13	26	435	15	85	588	87	4

VGG19	80–20%	244	0	12	216	3	43	295	53	1
	70–30%	360	1	24	331	17	59	438	71	6
	60–40%	476	9	38	580	87	12	443	31	77

VGG16	80–20%	243	1	13	212	5	4	296	54	0
	70–30%	361	4	23	343	9	47	440	61	4
	60–40%	466	11	46	386	4	134	592	165	0

## Data Availability

The data used to support the findings of this study are included within the article.
